# Ionic cellulose-stabilized gold nanoparticles and their application in the catalytic reduction of 4-nitrophenol

**DOI:** 10.1039/c7ra11393e

**Published:** 2018-01-08

**Authors:** J. Hwang, A. B. Siddique, Y. J. Kim, H. Lee, J. H. Maeng, Y. Ahn, J. S. Lee, H. S. Kim, H. Lee

**Affiliations:** Department of Chemistry and Research Institute of Basic Sciences, Kyung Hee University 26 Kyungheedaero Dongdaemun-gu Seoul 02447 Republic of Korea khs2004@khu.ac.kr; Clean Energy Center, Korea Institute of Science and Technology 5 Hwarang-ro 14-gil Sungbuk-gu Seoul 02792 Republic of Korea hjlee@kist.re.kr

## Abstract

A novel strategy for the synthesis of highly stable gold nanoparticles (GNPs) was designed by reducing HAuCl_4_ with NaBH_4_ in an aqueous solution of water-soluble ionic cellulose composed of dimethylimidazolium cations and phosphite-bound cellulose anions. NMR and UV-Vis analysis along with the measurement of the zeta potential suggest that the exceptionally high stability of GNPs originates from the strong interaction of GNPs with the phosphite groups of the ionic cellulose. The thus prepared GNPs exhibit excellent catalytic activity for the reduction of 4-nitrophenol to 4-aminophenol, a model hydrogenation reaction.

## Abstract 2

Gold nanoparticles (GNP) were highly stabilized by water soluble ionic cellulose by the strong interaction of GNP with the phosphite groups and showed extremely high catalytic activity for the reduction of 4-nitrophenol to 4-aminophenol.

## Graphical abstract

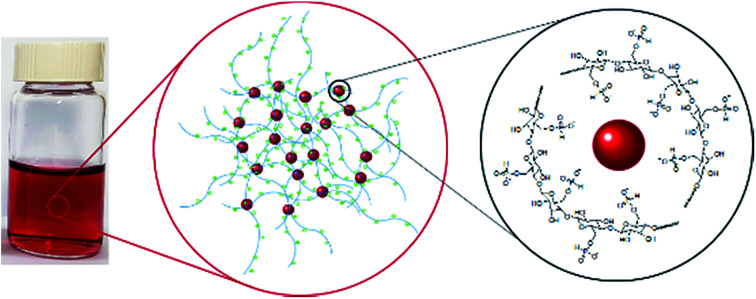

## 1. Introduction

Gold nanoparticles (GNPs) have attracted increasing interest due to their unique physicochemical properties, such as electrical, optical, and catalytic, that can be used in a wide variety of areas: chemical sensing,^1,2^ drug delivery systems,^3,4^ biomedicine,^5^ bioimaging,^6^ photothermal therapy,^7,8^ photothermal solar distillation,^9,10^ plasmonic photovoltaics,^11,12^ photocatalysis,^13–15^ and catalysis.^16–18^ GNPs can be easily prepared by reducing AuCl_3_ or HAuCl_4_ in an aqueous solution.^19,20^ However, GNPs prepared this way tend to easily agglomerate with time unless a suitable stabilizer is present.^21,22^ For the utilization of GNPs, their sizes should be kept constant during storage and application because desired properties for specific purposes are strongly affected by the sizes of the GNPs. To use GNPs, desired properties for specific purposes should not be impaired much during storage and application. This can be achieved by keeping the sizes and shapes of GNPs constant using suitable stabilizers. Much effort has been devoted to the stabilization of GNPs to suppress aggregation. Accordingly, numerous synthetic methods have been developed to prepare GNPs with enhanced stability.^23–28^ Nonetheless, much remains to be improved for the practical application of GNPs, especially in terms of long-term stability.

The recovery and reuse of costly GNPs from the reaction system is another real challenge. To this end, GNPs have been prepared in the presence of a solid support, such as polymers, carbon nanotubes, TiO_2_, and Al_2_O_3_.^29–32^ It has been reported that imidazolium-based ionic polymers can stabilize nanoparticles through electrostatic interactions.^33–36^ However, the strategy of using ionic polymers as stabilizers has some limitation due to the complexity of synthesizing ionic polymers. Recently, cellulose fibres and crystalline celluloses have also been employed as supports for GNPs, but the binding strength of GNPs with neutral celluloses remains questionable.^37,38^

We now report a green and convenient method for the synthesis of highly stabilized GNPs using water soluble ionic cellulose (IC) composed of dimethylimidazolium cations and phosphite (HPO_3_)-containing cellulose anions.^39^ The excellent catalytic performance of the ionic cellulose encapsulated GNPs is also demonstrated for the reduction of 4-nitrophenol in the presence of NaBH_4_ under ambient conditions.

## 2. Results and discussion

**Scheme 1:**
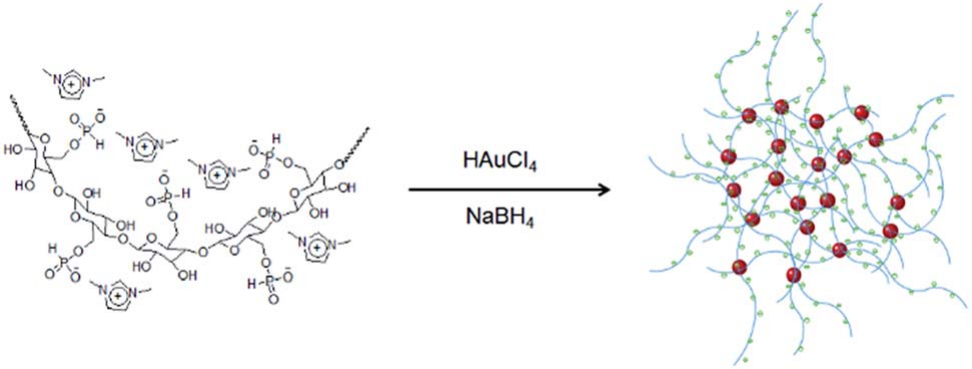
Synthesis of IC-GNPs.

An environmentally benign method was developed for the synthesis of highly dispersed and extremely stable GNPs using IC as shown in Scheme 1.

### 2.1 Synthesis of IC-GNPs

IC-GNPs were synthesized by treating an aqueous solution of HAuCl_4_ and IC (with various degrees of phosphorylation (DP) at around 1) with NaBH_4_ in the IC/Au molar ratio range of 5–100, and their morphology was investigated by UV-Vis, TEM, and XRD.

**Fig. 1:**
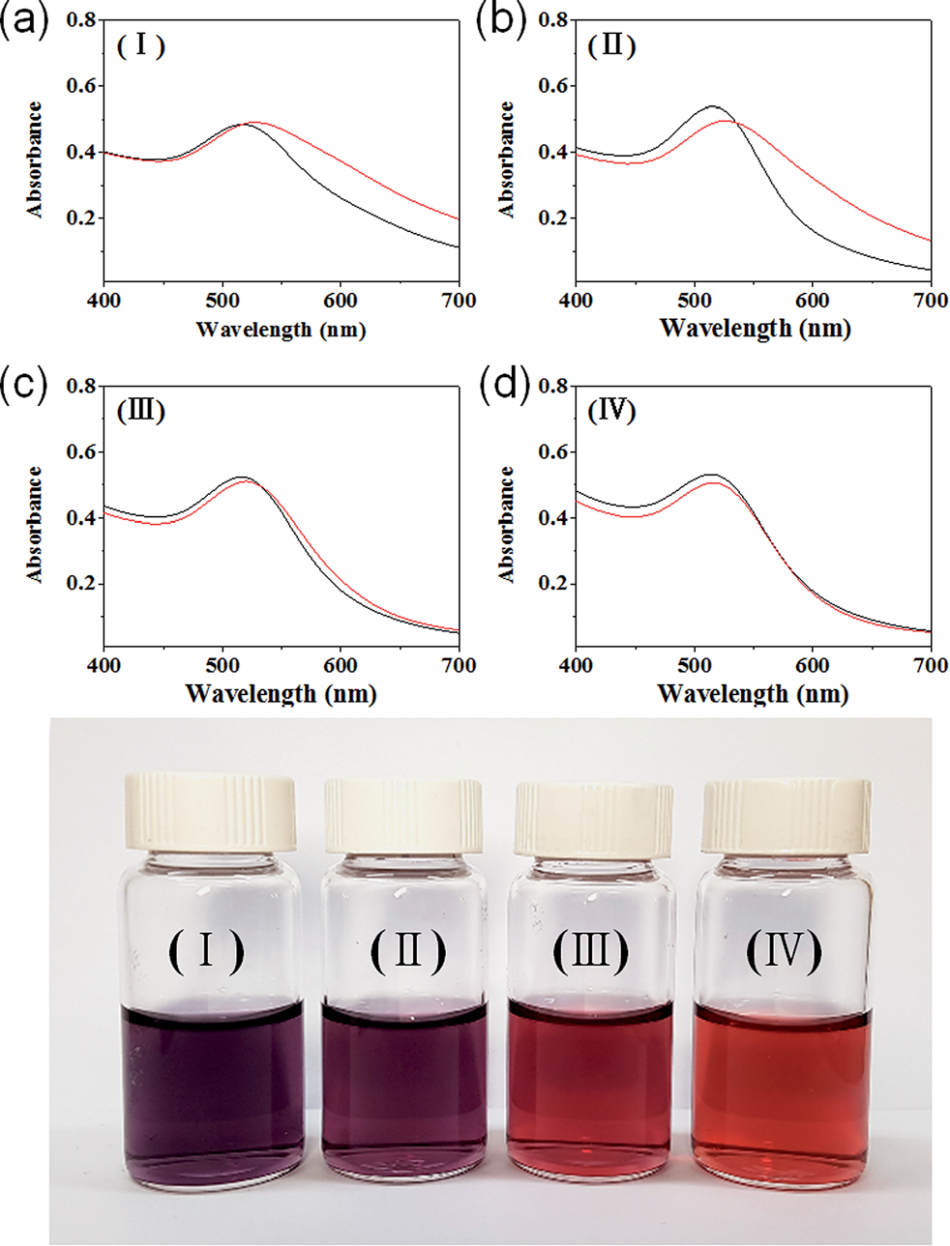
(Top) UV-Vis absorption spectra of the aqueous solutions of IC-GNPs prepared at various IC/Au molar ratios (DP = 1.09): (a) I (IC/Au = 15), (b) II (IC/Au = 25), (c) III (IC/Au = 50), (d) IV (IC/Au = 100); black line (30 min after synthesis), red line (one month after synthesis). (Bottom) Photographs of IC-GNP solutions (I–IV) taken one month after synthesis.

The colour of the solution containing HAuCl_4_ and IC changed from orange to wine red after the addition of NaBH_4_, indicating that GNPs with sizes below 10 nm were formed. However, as can be seen in Fig. 1, the colour of the resulting solution prepared at the molar ratios of 25 or lower turned to purple from wine red after being stored overnight. On the other hand, the wine red colour was persisted even after 1 month when a solution of GNPs was prepared at the IC/Au molar ratio of 50 or higher, demonstrating the outstanding performance of IC as a stabilizer for GNPs.

### 2.2 Characterization of IC-GNPs

The change in morphology and the stability of IC-GNPs (DP = 1.09) with different IC/Au molar ratios were investigated by UV-Vis spectroscopy and X-ray diffraction (XRD). As displayed in Fig. 1, all the aqueous solutions of IC-GNPs exhibited a characteristic surface-plasmon resonance absorption band centred at around 520 nm, supporting the formation of GNPs with a core diameter below 10 nm. However, the UV-Vis spectra taken 30 days after their synthesis clearly revealed that the long-term stability of the IC-GNPs was strongly dependent on the molar ratio of the IC/Au. The absorption bands at 520 nm were shifted to higher wavelengths for the GNPS prepared at the IC/Au molar ratios of 25 and lower, but the UV-Vis spectra of IC-GNPs obtained at the molar ratios of 50 and higher remained unchanged after one month storage at an ambient temperature. The effect of IC/Au on the stability of IC-GNPs is more evident from the colour change of the solutions of the IC-GNPs in water. As the photographs show, the red wine colour persisted for at least for 30 days for the aqueous solutions of IC-GNPs with the IC/Au molar ratios of 50 and 100, whereas the colours of the IC-GNPs solutions prepared at the IC/Au molar ratios of 15 and 25 turned to purple from red wine when stored for 30 days at an ambient temperature.

**Fig. 2:**
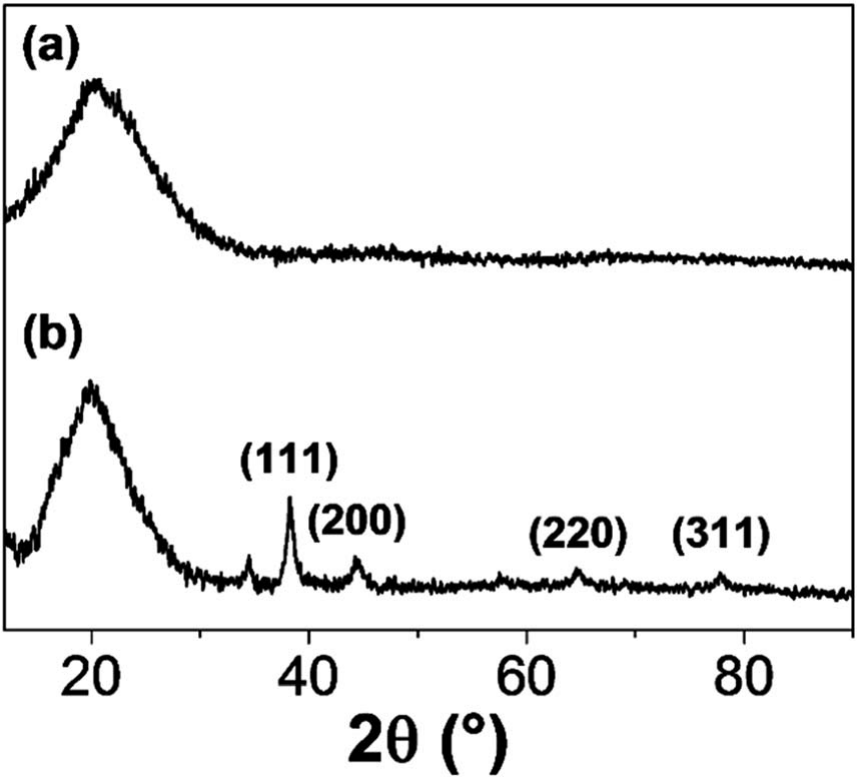
XRD patterns of solid IC (DP = 1.09) and IC-GNPs (III, molar ratio of IC/Au = 50): (a) IC and (b) IC-GNPs.

**Fig. 3:**
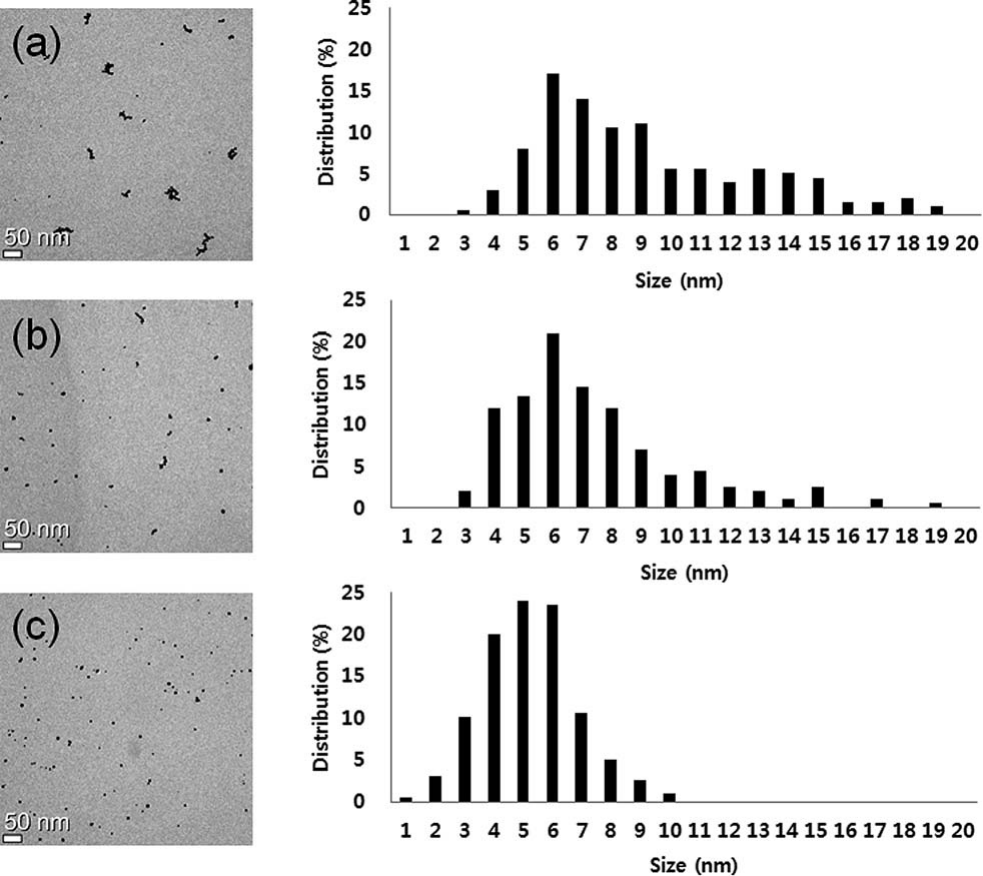
TEM images and corresponding histograms showing the size distribution of IC-GNPs (DP = 1.09): (a) I (IC/Au = 15), (b) II (IC/Au = 25), (c) III (IC/Au = 50).

The formation of GNPs was further confirmed by XRD analysis of the IC-GNPs prepared at the IC/Au molar of 50. As Fig. 2 reveals, IC-GNPs shows characteristic peaks of GNPs, which are not observed in the XRD patterns of IC. The peaks at 2*θ* = 38.3°, 44.3°, 64.7°, and 77.5° can be associated with the planes (111), (200), (220), and (311), respectively.^30^Fig. 3 shows TEM images and the corresponding histograms of particle size distribution of the aqueous solutions of IC-GNPs (DP = 1.09) prepared at the IC/Au molar ratios of 15, 25, and 50, respectively. The TEM images and histograms suggest that the particle size distribution of GNPs was strongly affected by the molar ratio of IC/Au: the higher the molar ratio, the narrower the size distribution. For the IC-GNP solution with the IC/Au molar ratio of 50, the size distribution was in the range of 1 to 10 nm. Such a narrow size distribution could be ascribed to the stabilizing effect of the IC, possibly through the interactions of phosphite groups of IC with GNPs.

The excellent stabilizing effect of IC for GNPs was supported by the extremely high zeta potential values of IC-GNPs approximately in the range of −40 to −62 (see Table 1). It is widely accepted that the zeta potential can be used as a key criterion indicator to evaluate the stability of nanoparticles because the magnitude of the zeta potential is a function of the degree of electrostatic repulsion between similarly charged adjacent particles in a dispersion medium.^40–42^ In this context, it is considered that the stability of nanoparticles with higher zeta potential possess higher stability, *i.e.*, the aggregation of nanoparticles is more resistant at higher zeta potential values. As listed in Table 1, IC-GNPs exhibited extremely high zeta potential values approximately in the range of −40 to −62, demonstrating the excellent stabilizing effect of IC for GNPs. The negative zeta potential increased with increasing degree of phosphorylation and the molar ratio of IC/Au. This strongly suggests that GNPs are surrounded by IC through interaction with the anionic phosphite groups.

**Table 1:** Zeta potentials of IC-GNPs prepared at various IC/Au molar ratios and DP values

IC/Au (molar ratio)	DP	Zeta potential
25	1.01	−40.5
	1.09	−46.5
	1.18	−51.0
50	1.01	−47.1
	1.09	−51.9
	1.18	−56.7
100	1.01	−49.6
	1.09	−57.7
	1.18	−62.1

**Fig. 4:**
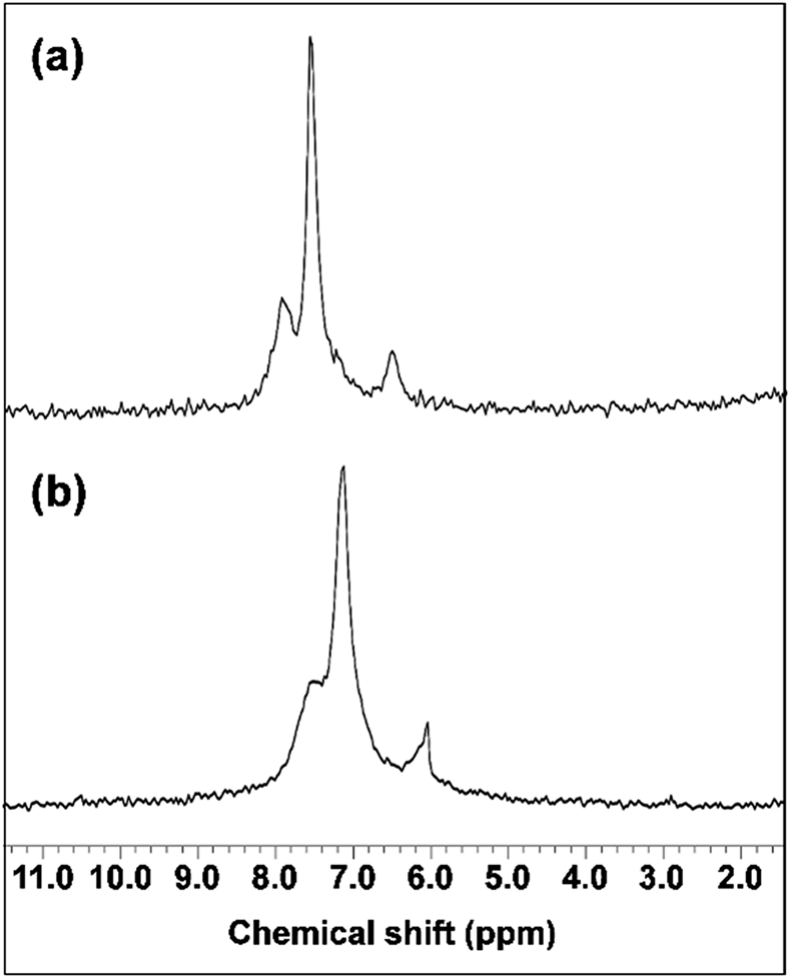
^31^P-NMR spectra: (a) IC, (b) IC-GNPs (molar ratio of IC/Au = 50, DP = 1.09).

**Scheme 2:**
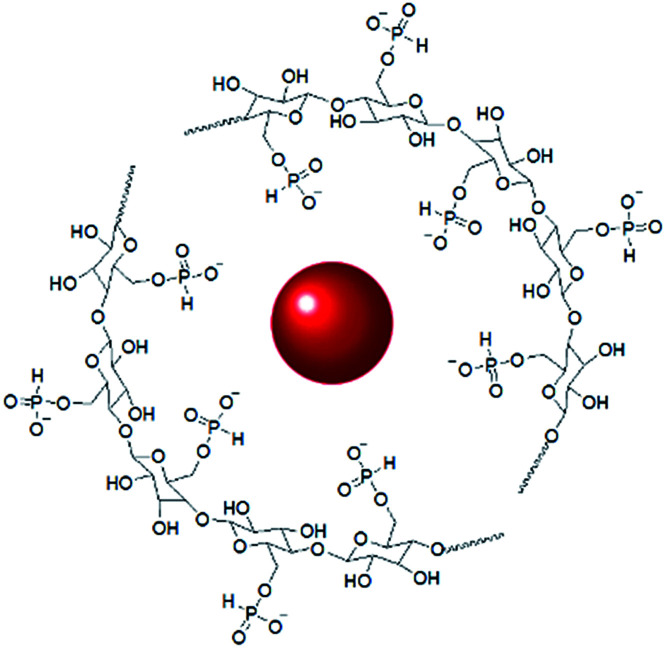
A scheme of IC-GNPs showing the interaction of GNP and anionic phosphite groups.

The interaction of GNPs with the phosphite groups bound to IC is somewhat supported by ^31^P NMR spectra of the IC (DP = 1.09) and IC-GNPs (DP = 1.09, molar ratio of IC/GNPs = 50). As can be seen in Fig. 4, the ^31^P NMR spectrum of the IC showed three peaks at 8.174, 7.592, and 6.705 ppm. Upon incorporation of GNPs, all three peaks were slightly shifted upfield to 7.564, 7.148, and 6.068 ppm, respectively. Considering the negative zeta potential and the ^31^P NMR results, it is evident that GNPs interact with the anionic phosphite groups of IC (see Scheme 2).

**Fig. 5:**
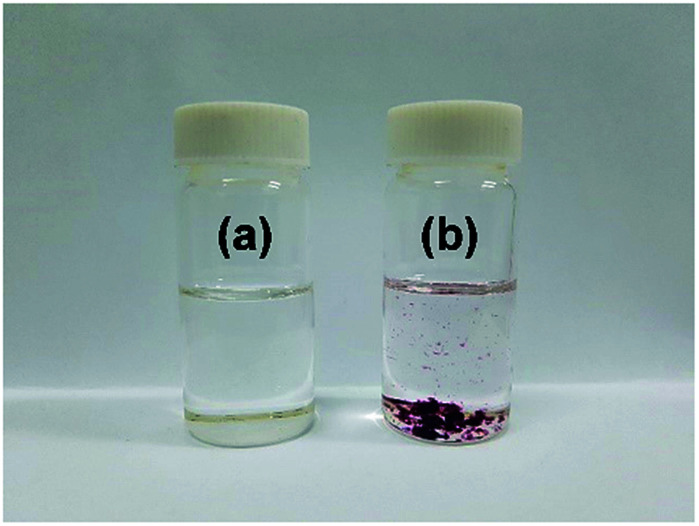
Photographs showing the different solubility of IC and IC-GNPs in DMSO: (a) IC and (b) IC-GNPs (IC/Au = 100, DP = 1.09).

A strong interaction between IC (DP = 1.09) and GNPs is further supported from the solubility difference of IC and IC-GNPs (DP = 1.09, molar ratio of IC/GNPs = 50) in dimethyl sulfoxide (DMSO). As shown in Fig. 5, IC was soluble in DMSO, forming a transparent solution. On the contrary, IC-GNPs were completely insoluble in DMSO.

### 2.3 Hydrogenation activities of IC-GNPs

Hydrogenation of 4-nitrophenol (4-NP) is of industrial importance because the product 4-aminophenol (4-AP) is commonly used as a developer for black-and-white film and as the final intermediate to paracetamol.^43^ The catalytic reduction of 4-NP to 4-AP using NaBH_4_ as a reducing agent is known to proceed through a redox mechanism accompanying electron transfer on nanoparticle surfaces.^27^ For this reason, the reduction of 4-NP is frequently employed as a probe reaction to evaluate the catalytic performance of stabilized GNPs.^44–46^

**Fig. 6:**
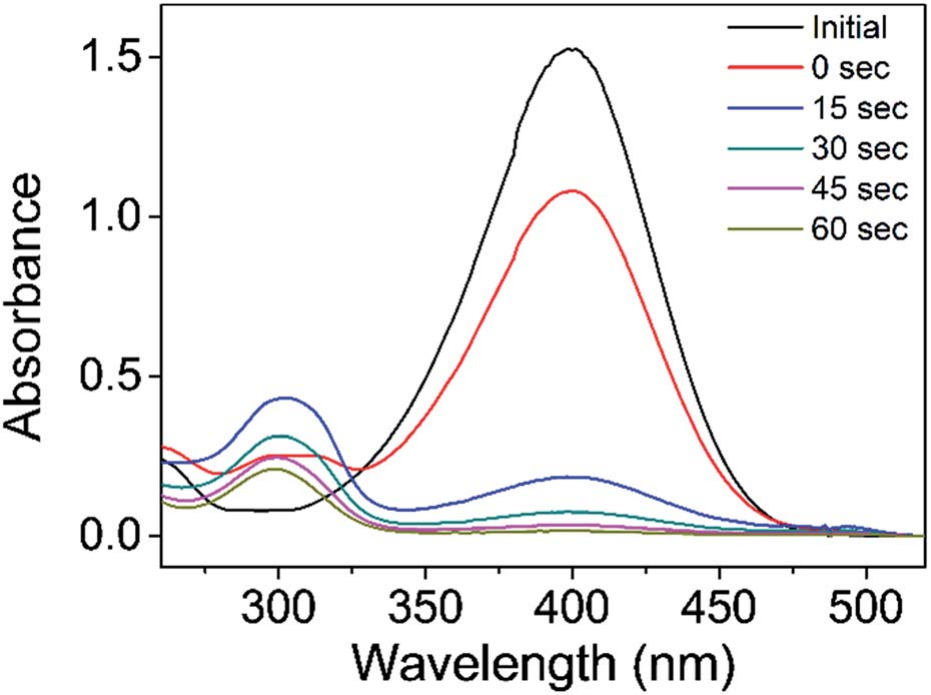
UV-Vis spectra showing the progress of the reduction of 4-NP to 4-AP catalysed by IC-GNPs (IC/Au = 100, DP = 1.09, NaBH_4_/Au = 80).

The catalytic reduction of 4-NP was conducted in a cuvette (3.5 mL) in the presence of IC-GNPs (molar ratio of IC/Au = 100, DP = 1.09) using NaBH_4_ as a reducing agent by varying the molar ratio of 4-NP/Au from 15 to 50, and the progress of the reaction was monitored by UV-Vis spectroscopy. As shown in Fig. 6, the intensity of the absorption peak at 400 nm corresponding to 4-nitrophenate, formed by the interaction of 4-NP with NaBH_4_, decreased with time along with the simultaneous appearance of the peak at 300 nm assignable to 4-AP. The reduction was fast and completed in about 1 min.

The rate of catalytic reduction of 4-NP was also investigated with the IC-GNPs (molar ratio of IC/Au = 100) by varying the DP and the molar ratio of 4-NP/Au from 15 to 50. As listed in Table 2, the rate of 4-NP reduction increased with increasing DP at the same 4-NP/Au molar ratio, implying that the stabilization of GNPs is extremely important in the reduction of 4-NP. As phosphite groups of IC are found to stabilize GNPs, it is obvious that IC-GNPs with higher DP exhibit higher activity for the reduction of 4-NP. The reduction rate became slower as 4-NP/Au molar ratio increased, but the rates of 4-AP formation were at least two times faster than those reported in the literature at all ranges of 4-NP/Au molar ratio.^44–47^ This strongly suggests that IC is more effective than the previously reported thiols, ionic polymers, and solid supports for the stabilization of GNPs. It is likely that IC-GNPs possess better redox properties than other stabilizers for the reduction of 4-NP using NaBH_4_ as a reducing agent.^27,48^ The rate of hydrogenation of 4-NP was not varied much with the molar ratio of NaBH_4_/Au in the range of 30–80, but increased drastically at much higher molar ratio at 2000. The hydrogenation of 4-NP did not take place at all in the absence of either NaBH_4_ or IC-GNPs (Table 2, entries 10 and 13).

**Table 2:** Pseudo first order rate constant for IC-GNPs (IC/Au = 100) catalysed reduction of 4-NP to 4-AP at varying DP and the molar ratios of 4-NP/Au and NaBH_4_/Au

Entry	Molar ratio (4-NP/Au)	Molar ratio (NaBH_4_/Au)	DP	*k* (s^−1^)	Ref.
1	15	80	1.01	0.0542	This work
2	15	80	1.09	0.0762	This work
3	15	80	1.18	0.0856	This work
4	30	80	1.01	0.0188	This work
5	30	80	1.09	0.0261	This work
6	30	80	1.18	0.0286	This work
7	50	80	1.01	0.007	This work
8	50	80	1.09	0.0076	This work
9	50	80	1.18	0.0092	This work
10	30	0	1.18	0	This work
11	30	30	1.18	0.0282	This work
12	30	50	1.18	0.0285	This work
13	No IC-GNP	80	1.18	0	This work
14	30	2000	1.18	0.0731	This work
15	28	2464	—	0.0128	43
17	5	44	—	0.0046	44
18	9.9	100	—	0.0122	45
19	11.7	200	—	0.006	46

### 2.4 Recyclability of IC-GNPs

The recycle test with the IC-GNPs (molar ratio of IC/Au = 100, DP = 1.09) for the reduction of 4-nitrophenol (4-NP). Molar ratios of 4-NP/Au and NaBH_4_/Au were set at 15 and 80, respectively. After 1 min of the reduction period, IC-GNPs were separated by filtration with the addition of acetone and used for further hydrogenation reaction at the same reaction condition. As can be seen in Table 3, the reduction rate of 4-NP to 4-aminophenol (4-AP) was found to decrease only by less than 3% after 5 cycles, demonstrating the excellent stability of IC-GNPs.

**Table 3:** Recycle test of IC/Au on the catalytic reduction of 4-NP to 4-AP

Recycle	*k* (s^−1^)
1	0.0753
2	0.0746
3	0.0741
4	0.0735
5	0.0731

## 3. Conclusion

Water-soluble ionic cellulose composed of dimethylimidazolium cations and phosphite-bound cellulose anions was highly efficient in stabilizing GNPs. The particle size of the GNPs and the color of the aqueous solutions of GNPs at the IC/Au molar ratios of 50 and higher remained unchanged even after 30 days from their preparation. ^31^P NMR spectral results, measurement of the zeta potential, and a solubility test suggested that the GNPs interacted strongly with the IC, most probably through the anionic phosphite groups. IC-GNPs showed extremely high catalytic activity for the reduction of 4-nitrophenol to 4-aminophenol.

## 4. Experimental section

### 4.1 Materials

All of the chemicals used for the synthesis of ionic cellulose-stabilized GNPs (IC-GNPs) were purchased from Aldrich Chemical Co. and used as received. Dimethylimidazolium methylphosphite [DMIm][(MeO)P(H)O_2_] was synthesized according to the literature procedure.^49^ IC-GNPs were prepared by reacting cellulose with [DMIm][(MeO)P(H)O_2_].

### 4.2 Synthesis of water soluble IC

Water-soluble ionic celluloses with various degrees of phosphorylation (DP) at around 1 were prepared by reacting cellulose (1 g) with [DMIm][(MeO)(H)PO_2_] (10 g) at 140 °C for 2–4 h.^39,50^ DP was considered as *x* value in eqn (1), which was derived from the structure of the repeating unit of phosphorylated cellulose, [C_6_H_7_O_2_(OH)_3−*x*_(HPO_3_)_*x*_][(C_5_H_9_N_2_)_*x*_] and phosphorous content obtained by elemental analysis.^50^

1 DP = (162 × % P)/[3100 − (161 × % P)]

where % P is the weight percentage of phosphorous in phosphorylated cellulose.

### 4.3 Preparation of IC-GNPs

A typical preparation of IC-GNPs is as follows. In a 100 mL 2-necked flask immersed in an ice bath, a solution of IC (0.08–0.64 g) in 40 mL of water was mixed with a 10 mL solution of HAuCl_4_ (1 mM). 0.5 mL of NaBH_4_ (0.05 M) was then added dropwise to the ice-cold solution containing HAuCl_4_ and IC. The yellow solution of HAuCl_4_ and IC turned wine-red immediately after the addition of NaBH_4_. The resulting solution was stirred further for 15 min and then stored at room temperature.

### 4.4 Characterization

IC-GNPs were characterized by UV-Vis spectroscopy (Agilent 8453), X-ray diffraction (PANalytical X'Pert PRO), transmission electron microscopy (TEM, FEI Tecnai F-20), and Zeta-seizer (Malvern Zetasizer Nano S).

### 4.5 Catalytic reduction of 4-nitrophenol

A stock solution was prepared by mixing a 3 mL solution of 4-nitrophenol (0.1 mM) in water with 0.4 mL aqueous solution of NaBH_4_ (0.1 wt%). Catalytic reduction of 4-nitrophenol was conducted in a cuvette in the presence of IC-GNPs with varying DP values.

### 4.6 Recycle test of IC-GNP

The catalytic reduction of 4-NP was carried out in a cuvette (3.5 mL) in the presence of IC-GNPs (molar ratio of IC/Au = 100, DP = 1.09) using NaBH_4_ as a reducing agent (molar ratio of NaBH_4_/Au = 80 and 4-NP/Au = 15), and the progress of the reaction was monitored by UV-Vis spectroscopy. After 1 min of the reduction period, IC-GNPs were separated by filtration with the addition of acetone and used for further hydrogenation reaction.

## Conflicts of interest

There are no conflicts to declare.

## Supplementary Material

## References

[cit1] Zeng S., Yong K.-T., Roy I., Dinh X.-Q., Yu X., Luan F. (2011). Plasmonics.

[cit2] Ma Y., Jiang L., Mei Y., Song R., Tian D., Huang H. (2013). Analyst.

[cit3] Cheng J., Gu Y.-J., Cheng S. H., Wong W.-T. (2013). J. Biomed. Nanotechnol..

[cit4] Daraee H., Eatemadi A., Abbasi E., Fekri Aval S., Kouhi M., Akbarzadeh A. (2016). Artif. Cells, Nanomed., Biotechnol..

[cit5] Palmal S., Jana N. R. (2014). Wiley Interdiscip. Rev.: Nanomed. Nanobiotechnol..

[cit6] Wani I. A. , Integrating Biologically-Inspired Nanotechnology into Medical Practice, ed. B. K. Nayak, A. Nanda and M. A. Bhat, IGI Global, Hershey, 2017

[cit7] Fazal S., Jayasree A., Sasidharan S., Koyakutty M., Nair S. V., Menon D. (2014). ACS Appl. Mater. Interfaces.

[cit8] Iodice C., Cervadoro A., Palange A., Key J., Aryal S., Ramirez M. R., Mattu C., Ciardelli G., O'Neill B. E., Decuzzi P. (2016). Optic Laser. Eng..

[cit9] Neumann O., Urban A. S., Day J., Lal S., Nordlander Pl., Halas N. J. (2012). ACS Nano.

[cit10] Fang Z., Zhen Y.-R., Neumann O., Polman A., Garciía de Abajo F. J., Nordlander P., Halas N. J. (2013). Nano Lett..

[cit11] Su Y.-H., Ke Y.-F., Cai S.-L., Yao Q.-Y. (2012). Light: Sci. Appl..

[cit12] Li Y., Wang H., Feng Q., Zhou G., Wang Z.-S. (2013). Energy Environ. Sci..

[cit13] Gomes Silva C. U., Juárez R., Marino T., Molinari R., García H. (2010). J. Am. Chem. Soc..

[cit14] Ke X., Zhang X., Zhao J., Sarina S., Barry J., Zhu H. (2013). Green Chem..

[cit15] Neatu S. T., Maciá-Agulló J. A., Concepción P., García H. (2014). J. Am. Chem. Soc..

[cit16] Daniel M.-C., Astruc D. (2004). Chem. Rev..

[cit17] Lam E., Hrapovic S., Majid E., Chong J. H., Luong J. H. (2012). Nanoscale.

[cit18] Mikami Y., Dhakshinamoorthy A., Alvaro M., García H. (2013). Catal. Sci. Technol..

[cit19] Agarwal S. V., Reddy S. S., Dhayal M. (2014). RSC Adv..

[cit20] Das A., Chadha R., Maiti N., Kapoor S. (2014). J. Nanopart..

[cit21] Deraedt C., Salmon L., Gatard S., Ciganda R., Hernandez R., Ruiz J., Astruc D. (2014). Chem. Commun..

[cit22] Dzimitrowicz A., Jamroz P., Greda K., Nowak P., Nyk M., Pohl P. (2015). J. Nanopart. Res..

[cit23] Oh E., Susumu K., Goswami R., Mattoussi H. (2010). Langmuir.

[cit24] Gan P. P., Ng S. H., Huang Y., Li S. F. Y. (2012). Bioresour. Technol..

[cit25] Malhotra A., Dolma K., Kaur N., Rathore Y. S., Mayilraj A. S., Choudhury A. R. (2013). Bioresour. Technol..

[cit26] Shim Y. B. (2013). J. Nanopart..

[cit27] Mishra A., Kumari M., Pandey S., Chaudhry V., Gupta K. C., Nautiyal C. S. (2014). Bioresour. Technol..

[cit28] Shivhare A., Ambrose S. J., Zhang H., Purves R. W., Scott R. W. J. (2013). Chem. Commun..

[cit29] Kim B., Sigmund W. M. (2004). Langmuir.

[cit30] Xu L.-X., He C.-H., Zhu M.-Q., Fang S. (2007). Catal. Lett..

[cit31] Thakor A., Jokerst J., Zavaleta C., Massoud T., Gambhir S. (2011). Nano Lett..

[cit32] Adnan R. H., Andersson G. G., Polson M. I., Metha G. F., Golovko V. B. (2015). Catal. Sci. Technol..

[cit33] Zhao D., Fei Z., Ang W. H., Dyson P. J. (2006). Small.

[cit34] Khalilzadeh M. A., Borzoo M. (2016). J. Food Drug Anal..

[cit35] Nikahd B., Khalilzadeh M. A. (2016). J. Mol. Liquids.

[cit36] Khalilzadeh M. A., Arab Z. (2017). Curr. Anal. Chem..

[cit37] Uryupina O. Y., Vysotskii V., Matveev V., Gusel'nikova A., Roldunghin V. (2011). Colloid J..

[cit38] Islam M. T., Padilla J. E., Dominguez N., Alvarado D. C., Alam M. S., Cooke P., Tecklenburg M. M., Noveron J. C. (2016). RSC Adv..

[cit39] Vo H. T., Kim Y. J., Jeon E. H., Kim C. S., Kim H. S., Lee H. (2012). Chem.–Eur. J..

[cit40] Greenwood R., Kendall K. (1999). J. Eur. Ceram. Soc..

[cit41] Kirby B. J. , Micro- and Nanoscale Fluid Mechanics: Transport in Microfluidic Devices, Cambridge University Press, New York, 2010

[cit42] Hanaor D., Michelazzi M., Leonelli C., Sorrell C. C. (2012). J. Eur. Ceram. Soc..

[cit43] Mitchell S. C. and Waring R. H., Aminophenols in Ullmann's Encyclopedia of Industrial Chemistry, Wiley-VCH Verlag GmbH & Co. KGaA, 2000

[cit44] Biondi I., Laurenczy G., Dyson P. J. (2011). Inorg. Chem..

[cit45] Huang T., Meng F., Qi L. (2009). J. Phys. Chem. C.

[cit46] Das S. K., Dickinson C., Lafir F., Brougham D. F., Marsili E. (2012). Green Chem..

[cit47] Gao Z., Su R., Huang R., Qi W., He Z. (2014). Nanoscale Res. Lett..

[cit48] Pradhan N., Pal A., Pal T. (2001). Langmuir.

[cit49] Palgunadi J., Kang J. E., Cheong M., Kim H., Lee H., Kim H. S. (2009). Bull. Korean Chem. Soc..

[cit50] Suflet D. M., Chitanu G. C., Popa V. I. (2006). React. Funct. Polym..

